# Supplementary figures and images for: Prolonged secondary hyperparathyroidism in adenine-induced CKD leads to skeletal changes consistent with skeletal hyporesponsiveness to PTH
Source: PLoS One. 2025 May 23;20(5):e0324628. doi: 10.1371/journal.pone.0324628 (PMC12101686; doi:10.1371/journal.pone.0324628)

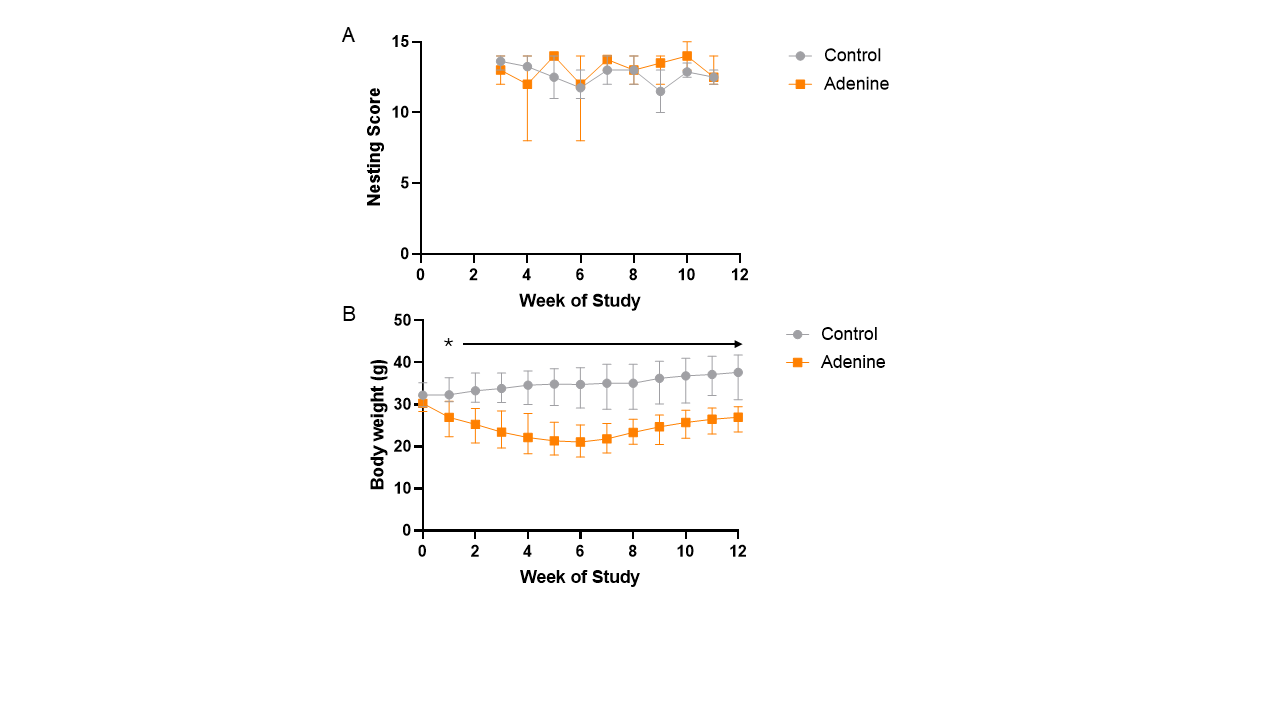

Supplement: S1 Fig — A) Cage nesting scores recorded during weeks 3–12 of the study. B) Body weights from baseline through week 12 of the study. *Indicates difference in body weight between control and adenine beginning after one week on the adenine diet. (TIF) [file pone.0324628.s001.tif]
