# Supplementary material for: Prolonged secondary hyperparathyroidism in adenine-induced CKD leads to skeletal changes consistent with skeletal hyporesponsiveness to PTH
Source: PLoS One. 2025 May 23;20(5):e0324628. doi: 10.1371/journal.pone.0324628 (PMC12101686; doi:10.1371/journal.pone.0324628)
Supplement: Attachment — Submitted filename: Response to reviewers.docx [file pone.0324628.s006.docx]

Journal Requirements:

When submitting your revision, we need you to address these additional requirements.

1. Please ensure that your manuscript meets PLOS ONE's style requirements, including those for file naming. The PLOS ONE style templates can be found at <https://journals.plos.org/plosone/s/file?id=wjVg/PLOSOne_formatting_sample_main_body.pdf> and <https://journals.plos.org/plosone/s/file?id=ba62/PLOSOne_formatting_sample_title_authors_affiliations.pdf>

Response: We have worked through the manuscript to address these formatting issues.

2. To comply with PLOS ONE submissions requirements, in your Methods section, please provide additional information regarding the experiments involving animals and ensure you have included details on (1) methods of sacrifice, (2) methods of anesthesia and/or analgesia, and (3) efforts to alleviate suffering.

Response: More details have been added on the anesthesia used during euthanasia, the euthanasia methods, and the general monitoring of the mice to ensure no suffering. No analgesia was needed nor any other supportive measures throughout the study. These details have been added.

3. Thank you for stating the following in the Acknowledgments Section of your manuscript:
This study was funded by the Department of Veterans Affairs via grants I01BX003025 and IK6-BX006479 awarded to MRA. Histological processing was completed through the Histology Lab Core Service in the Indiana Center for Musculoskeletal Health (P30-AR083854 to AG Robling).

We note that you have provided funding information that is not currently declared in your Funding Statement. However, funding information should not appear in the Acknowledgments section or other areas of your manuscript. We will only publish funding information present in the Funding Statement section of the online submission form.
Please remove any funding-related text from the manuscript and let us know how you would like to update your Funding Statement. Currently, your Funding Statement reads as follows:
This study was funded by the Department of Veterans Affairs via grants I01BX003025 and IK6-BX006479 awarded to MRA.

Please include your amended statements within your cover letter; we will change the online submission form on your behalf.

Response: This has been removed from the text of the manuscript. The revised statement is included in the cover letter.

4. [Please include captions for your Supporting Information files at the end of your manuscript, and update any in-text citations to match accordingly. Please see our Supporting Information guidelines for more information: http://journals.plos.org/plosone/s/supporting-information.](https://nam12.safelinks.protection.outlook.com/?url=http%3A%2F%2Ftrack.editorialmanager.com%2FCL0%2Fhttp%3A%252F%252Fjournals.plos.org%252Fplosone%252Fs%252Fsupporting-information.%2F1%2F010f019547f8640c-ca175ac8-0a1e-4581-ac34-fb1d858b0ddc-000000%2FnqZflpCg85oFe9RH7uc2GvQ9ZlKLn3rzbk0NJpTpDYg%3D200&data=05%7C02%7Ccormetzg%40iu.edu%7Cb5a1f77d110e4a2b1bc808dd57768290%7C1113be34aed14d00ab4bcdd02510be91%7C1%7C0%7C638762888684961097%7CUnknown%7CTWFpbGZsb3d8eyJFbXB0eU1hcGkiOnRydWUsIlYiOiIwLjAuMDAwMCIsIlAiOiJXaW4zMiIsIkFOIjoiTWFpbCIsIldUIjoyfQ%3D%3D%7C0%7C%7C%7C&sdata=zt8fauYgKTOwb2NFY7IX%2BO1cUAOWSVdEE%2FYVOsqJNME%3D&reserved=0)

Response: This has been completed.

5. Please review your reference list to ensure that it is complete and correct. If you have cited papers that have been retracted, please include the rationale for doing so in the manuscript text, or remove these references and replace them with relevant current references. Any changes to the reference list should be mentioned in the rebuttal letter that accompanies your revised manuscript. If you need to cite a retracted article, indicate the article’s retracted status in the References list and also include a citation and full reference for the retraction notice.

Response: All citations were searched by article title or DOI on the Retraction Watch Database and no retractions were found. References with available doi were updated to fit journal requirements.


[Note: HTML markup is below. Please do not edit.]

Reviewers' comments:

Reviewer's Responses to Questions

**Comments to the Author**

1. Is the manuscript technically sound, and do the data support the conclusions?

The manuscript must describe a technically sound piece of scientific research with data that supports the conclusions. Experiments must have been conducted rigorously, with appropriate controls, replication, and sample sizes. The conclusions must be drawn appropriately based on the data presented.

Reviewer #1: Partly

Reviewer #2: Yes

2. Has the statistical analysis been performed appropriately and rigorously?

Reviewer #1: Yes

Reviewer #2: Yes

3. Have the authors made all data underlying the findings in their manuscript fully available?

The [PLOS Data policy](https://nam12.safelinks.protection.outlook.com/?url=http%3A%2F%2Ftrack.editorialmanager.com%2FCL0%2Fhttp%3A%252F%252Fwww.plosone.org%252Fstatic%252Fpolicies.action%2523sharing%2F1%2F010f019547f8640c-ca175ac8-0a1e-4581-ac34-fb1d858b0ddc-000000%2F-sxwETU5VpnQK_Ovx0--zLB1f1us8uoTxheBW6TKmS0%3D200&data=05%7C02%7Ccormetzg%40iu.edu%7Cb5a1f77d110e4a2b1bc808dd57768290%7C1113be34aed14d00ab4bcdd02510be91%7C1%7C0%7C638762888684975596%7CUnknown%7CTWFpbGZsb3d8eyJFbXB0eU1hcGkiOnRydWUsIlYiOiIwLjAuMDAwMCIsIlAiOiJXaW4zMiIsIkFOIjoiTWFpbCIsIldUIjoyfQ%3D%3D%7C0%7C%7C%7C&sdata=kBc94yLKFwNLoDuIFuU8ItsYWKy3rajC%2FF49Gyez3pE%3D&reserved=0) requires authors to make all data underlying the findings described in their manuscript fully available without restriction, with rare exception (please refer to the Data Availability Statement in the manuscript PDF file). The data should be provided as part of the manuscript or its supporting information, or deposited to a public repository. For example, in addition to summary statistics, the data points behind means, medians and variance measures should be available. If there are restrictions on publicly sharing data—e.g. participant privacy or use of data from a third party—those must be specified.

Reviewer #1: Yes

Reviewer #2: Yes

4. Is the manuscript presented in an intelligible fashion and written in standard English?

PLOS ONE does not copyedit accepted manuscripts, so the language in submitted articles must be clear, correct, and unambiguous. Any typographical or grammatical errors should be corrected at revision, so please note any specific errors here.

Reviewer #1: Yes

Reviewer #2: Yes

5. Review Comments to the Author

Please use the space provided to explain your answers to the questions above. You may also include additional comments for the author, including concerns about dual publication, research ethics, or publication ethics. (Please upload your review as an attachment if it exceeds 20,000 characters)

Reviewer #1: The longitudinal design of the study is a notable strength, as it captures the progression of skeletal changes over time—a feature often lacking in animal models of CKD.

However, there are several areas where the study could be improved. First, the exclusive use of male mice limits the generalizability of the findings, given the well-documented sex-specific differences in bone metabolism and CKD progression. Including female mice in future studies would provide a more comprehensive understanding of these dynamics. Second, while the study identifies altered osteocyte characteristics and reduced expression of PTH receptor 1 (PTHR1) and receptor activator of nuclear factor κB ligand (RANKL) as potential mechanisms, it does not explore the role of other CKD-related factors, such as uremic toxins or systemic inflammation, which may also contribute to skeletal hypo responsiveness.

Response: Thank you for your comments. In the limitations paragraph we addressed the limitation of only assessing male mice as well as the need to address uremic toxins and their role in future studies with citations of in vitro work on that front. We have now also included a sentence addressing the unexplored role of other factors like systemic inflammation and oxidative stress.

Reviewer #2: The manuscript entitled "Prolonged secondary hyperparathyroidism in adenine-induced CKD leads to skeletal
changes consistent with skeletal hyporesponsiveness to PTH" have designed and written well. No any major correction and grammatical mistake reported at present as per my view. The present manuscript can be accepted for publication.

Response: Thank you for your review.

6. PLOS authors have the option to publish the peer review history of their article ([what does this mean?](https://nam12.safelinks.protection.outlook.com/?url=https%3A%2F%2Ftrack.editorialmanager.com%2FCL0%2Fhttps%3A%252F%252Fjournals.plos.org%252Fplosone%252Fs%252Feditorial-and-peer-review-process%2523loc-peer-review-history%2F1%2F010f019547f8640c-ca175ac8-0a1e-4581-ac34-fb1d858b0ddc-000000%2F2Gzn_4SjTOkQcs-2dRNjwbRGQqz8CPR27iCB-5eoQg0%3D200&data=05%7C02%7Ccormetzg%40iu.edu%7Cb5a1f77d110e4a2b1bc808dd57768290%7C1113be34aed14d00ab4bcdd02510be91%7C1%7C0%7C638762888684989821%7CUnknown%7CTWFpbGZsb3d8eyJFbXB0eU1hcGkiOnRydWUsIlYiOiIwLjAuMDAwMCIsIlAiOiJXaW4zMiIsIkFOIjoiTWFpbCIsIldUIjoyfQ%3D%3D%7C0%7C%7C%7C&sdata=UGwZbiKXOs39lOQljR4dCy9qJ3WqMsqmNYZMmBKYuXI%3D&reserved=0)). If published, this will include your full peer review and any attached files.


If you choose “no”, your identity will remain anonymous but your review may still be made public.


**Do you want your identity to be public for this peer review?** For information about this choice, including consent withdrawal, please see our [Privacy Policy](https://nam12.safelinks.protection.outlook.com/?url=https%3A%2F%2Ftrack.editorialmanager.com%2FCL0%2Fhttps%3A%252F%252Fwww.plos.org%252Fprivacy-policy%2F1%2F010f019547f8640c-ca175ac8-0a1e-4581-ac34-fb1d858b0ddc-000000%2FAfj-eCqp1Vd7O1pypIpGwiuaMAFyeie1B8JMRL-moDo%3D200&data=05%7C02%7Ccormetzg%40iu.edu%7Cb5a1f77d110e4a2b1bc808dd57768290%7C1113be34aed14d00ab4bcdd02510be91%7C1%7C0%7C638762888685006793%7CUnknown%7CTWFpbGZsb3d8eyJFbXB0eU1hcGkiOnRydWUsIlYiOiIwLjAuMDAwMCIsIlAiOiJXaW4zMiIsIkFOIjoiTWFpbCIsIldUIjoyfQ%3D%3D%7C0%7C%7C%7C&sdata=QjZXPdH5sM4XHBynQJ5NKyhIA3QdK%2FLLXFLWPEzIklU%3D&reserved=0).

Reviewer #1: **Yes:** Dr.Zena A.A.Hadedy

Reviewer #2: No
